# Unique Transcompartmental Bridge: Antigen-Presenting Cells Sampling across Endothelial and Mucosal Barriers

**DOI:** 10.3389/fimmu.2016.00231

**Published:** 2016-06-10

**Authors:** Frederick Allen, Alexander A. Tong, Alex Y. Huang

**Affiliations:** ^1^Department of Pathology, Case Western Reserve University School of Medicine, Cleveland, OH, USA; ^2^Department of Pediatrics, Case Western Reserve University School of Medicine, Cleveland, OH, USA; ^3^Angie Fowler AYA Cancer Institute, University Hospitals Rainbow Babies & Children’s Hospital, Cleveland, OH, USA

**Keywords:** dendritic cell, antigen-presenting cell, mucosal immunity, transcompartment, T cell activation, antigen presentation, cell migration, antigen capture

## Abstract

Potentially harmful pathogens can gain access to tissues and organ systems through body sites that are in direct contact with the outside environment, such as the skin, the gut, and the airway mucosa. Antigen-presenting cells (APCs) represent a bridge between the innate and adaptive immunity, and their capacity for constant immune surveillance and rapid sampling of incoming pathogens and other potentially harmful antigens is central for mounting an effective and robust protective host response. The classical view is that APCs perform this task efficiently within the tissue to sense invading agents intra-compartmentally. However, recent data based on high resolution imaging support an additional transcompartmental surveillance behavior by APC by reaching across intact physical barriers. In this review, we summarize intravital microscopic evidences of APC to sample antigens transcompartmentally at the gut mucosa and other body sites.

## Introduction

Our immune system is tasked to develop host defense mechanisms that require speed, sensitivity, and specificity to deal with a vast array of fast evolving pathogens, since the doubling times of bacteria and viruses outpace eukaryotic cell division in most cases. In this regard, current dogma for the initiation of the adaptive cellular immune responses has centered primarily on the role of tissue-resident dendritic cells (DCs) and their ability to migrate to the draining lymph nodes (DLNs) and present processed foreign antigens bound to surface major histocompatibility complexes (MHC) to T cells for recognition *via* cognate T cell receptor (TCR). However, DC maturation upon pathogen encounter and its subsequent migration to LN can take hours to days. Upon arrival in the LN, antigen-laden DCs’ presentation to and engagement by T cells can take significant time to mount an effective antigen-specific cellular immune response. Emerging data suggest an alternative route of antigen capture and presentation by DCs and other antigen-presenting cells (APCs) across distinct anatomical compartments. For example, data now suggest that crosstalk between cells in the sinuses and those located within the parenchyma is essential for the maintenance of immune cells during steady (tolerant-inducing) and non-steady (inflammatory-inducing) states in the LN (1–3). Although immune cell transmigration and shuffling of antigens across endothelial and epithelial conduits is not new, the concept that APCs residing in one tissue compartment can extend their processes across intact epithelial and endothelial barriers into sites such as endothelial vessels, LN conduits, airway passages, gut lumen, and central nervous system (CNS) vasculature without physical transmigration is a growing area of research with potential biological significance. This new appreciation of APC behavior *in vivo* is made possible due to growing and increasingly sophisticated application of the intravital two-photon laser scanning microscopy (2PLSM) technique in anesthetized mice or intact explanted tissues. Knowledge gained from the growing body of investigations adds to our new understanding of how the immune system mounts meaningful and timely responses to infections and other pathologies and is poised to impact the development of novel vaccine strategies. This review focuses on a presentation of available intravital imaging-based evidence regarding the transcompartmental behavior of DCs and other APCs in various body sites. We will first discuss APC behavior in the gut mucosa, ending with a discussion on the intra-nodal APC behavior in the sentinel LN and other anatomical sites including the airways and the CNS.

## Gut Mucosa: APC Transepithelial Extensions in Lamina Propria and Peyer’s Patch

The gut mucosa is constantly exposed to commensal and potentially pathogenic microbial antigens. Historically, antigen has been thought to be primarily taken up through M cells in the Peyer’s patch (PP) for presentation to APCs on the basolateral side of the mucosal barrier (4–6). These APCs were then thought to process and present this antigen in the draining mesenteric LN, thereby initiating a gut-trophic adaptive immune response. However, multiple studies using *in situ* microscopic evaluation of intestinal lymphoid structures suggest that there may be active surveillance of the brush border *via* APCs extending dendrites between epithelial cells in order to directly sample luminal antigen. These data reveal that there may be an additional, complex layer of immune surveillance in the form of transepithelial/transcompartmental extension (TE) at the host–pathogen barrier, which allows efficient antigen uptake and processing.

For the purposes of this review, we will generally define gut resident DCs as CD11c^+^CX3CR1^−^CD103^+^ cells and gut resident macrophages (Macs) as CD11c^+^CX3CR1^+^CD103^−^ cells, although we recognize that there may be many more nuanced subsets depending on the markers used (7–12). Due to the more recent nature of these combinations of markers being used to discriminate the two populations, cell-type identifications from older literature reviewed hereafter will be indicated as clearly as the data allow.

Some of the first studies utilizing *in vitro* and limited *in vivo* approaches focused on DC extensions through intact epithelial barrier in the lamina propria (LP) (13). The earliest studies used transwell coated with human enterocyte cell line Caco-2 to establish an *in vitro* model of the gut epithelium. DCs were then seeded on the basolateral side with bacteria added to the luminal side. Electron and confocal microscopy of this system revealed that DCs were able to penetrate the Caco-2 monolayer to directly uptake bacteria. Complementary immunostaining studies using ligated loops of intestine injected with non-infective *Escherichia coli* DH5α or infective *Salmonella typhimurium* showed that some CD11c^+^ DCs were able to extend dendrites into the lumen and uptake infective bacteria. Importantly, these DCs expressed tight junction proteins of their own, suggesting that they were able to maintain brush border microvilli organization *via* contiguous connection with epithelial cells.

Subsequently, Niess et al. used living intestinal explants to identify CX3CR1^+^ APCs specifically in the terminal ileum extending dendrites into the bowel lumen (14). This explant system revealed that the number of LP APCs extending dendrites per villus ranged from ~1–2 in the non-inflamed setting to ~4 in the *Salmonella* infected setting. Here, LP APCs were dependent on recognition of CX3CL1 on intestinal epithelial cells (IECs) for their function, as only CX3CR1^GFP/WT^ (functional heterozygous) but not CX3CR1^GFP/GFP^ (knockout homozygous) animals were able to extend luminal dendrites.

Other groups adapted the intestinal explant technique for 2PLSM visualizing the luminal side of the LP. Chieppa et al. everted clips of small intestine from various reporter mice to expose the lumen for 2PLSM (15). They showed that LP APCs project extensions into gut lumen with either finger-like projections or a distinct balloon body (BB) shape in order to sample pathogenic *S. typhimurium*. As the BB projections were not visualized in other explant preparations, the authors acknowledged that it could be an artifact secondary to removal of the mucus layer, although other data suggest that these could be APCs beginning to unidirectionally transmigrate into the gut lumen in a flagellin-dependent manner in order to participate in pathogen exclusion (16). Significantly, in contrast to previous studies, Chieppa et al. found that CD11c^+^/MHC-II^+^ APC extensions into lumen were visualized in all parts of the small intestine LP at densities similar to previous reports (~1–5 DCs per villus), raising the question whether this mode of antigen uptake could be more universally distributed than previously described (14, 15). A later study by Hapfelmeier et al. examined specifically the large intestine LP DCs using the same explant approach and failed to identify any TEs in the cecum (17). However, cecal LP DCs were still found to be critical for facilitating the early phase of *S. typhimurium* infection, and the authors did not rule out the possibility that rare LP DC sampling events not captured by imaging could drive *Salmonella* epithelial translocation.

The ability of gut APCs to extend TEs into lumen is not restricted to LP APCs. Lelouard et al. used 2PLSM of PP explants to identify highly mobile PP CD11c^+^ APCs extending dendrites through M-cell specific pores (18). The number of APCs with trans-M cell dendrites (TMDs) was estimated to be 35–125 per PP, and these TMDs could actively retract back into the sub-epithelial dome of the PP after sampling the lumen. Consistent with LP APC studies, PP APCs were able to uptake *S. typhimurium* through TMDs in the early hours of infection.

All of the explant studies discussed above involve significant trauma to the intestinal explant (lengthwise incision followed by multiple washes) or ligated loops. Therefore, more recent studies have taken a relatively less invasive, intravital approach to visualizing LP APCs (19, 20). Laboratories have accomplished this by making a small abdominal incision, drawing out a small portion of the small intestine and securing this to a coverslip by epoxy or by the mouse’s own weight for imaging on an inverted microscope. Using a serosal 2PLSM imaging approach, McDole et al. showed that goblet cells deliver low molecular weight antigen to CD103^+^ DCs in the small intestine, whereas CX3CR1^+^ Macs may directly sample higher molecular weight antigen *via* extensions into gut lumen (19). In a similar fashion through a mucosal imaging approach, Xu et al. demonstrated that CX3CR1^+^ Macs project into LP *in vivo*, although these Macs are largely sessile in the uninflamed setting and do not appear to migrate away from the epithelium (20). In contrast, a subsequent intravital study by Farache et al. failed to demonstrate CX3CR1^+^ Macs exhibiting TEs even under pathogenic bacterial stimulation. Instead, CD103^+^CX3CR1^−^ DCs were recruited to the epithelium by *S. typhimurium*, thereafter extending TEs to directly sample and capture luminal bacteria (21).

While there is strong evidence for APC TEs based on the above imaging studies, there are contrasting data questioning the universality of these observations. Ligated small intestinal loop studies using a BALB/c mouse background strain and a model fungal pathogen failed to show the same dendrite formation (22). Depletion of CD11c^+^ cells still allowed for fungal pathogen translocation into LP in this *in vitro* infection model, suggesting that LP DCs or Macs were not critical for pathogen uptake. A comparison of CX3CR1 reporter mice on BALB/c, C57BL/6, and F1 backgrounds revealed that as-of-yet undefined genetic factors in BALB/c mice may be responsible for the absence of TEs originally identified in C57BL/6 strains. The outstanding question remains whether these APC extensions are relevant in human mucosal surface surveillance.

**Figure 1:**
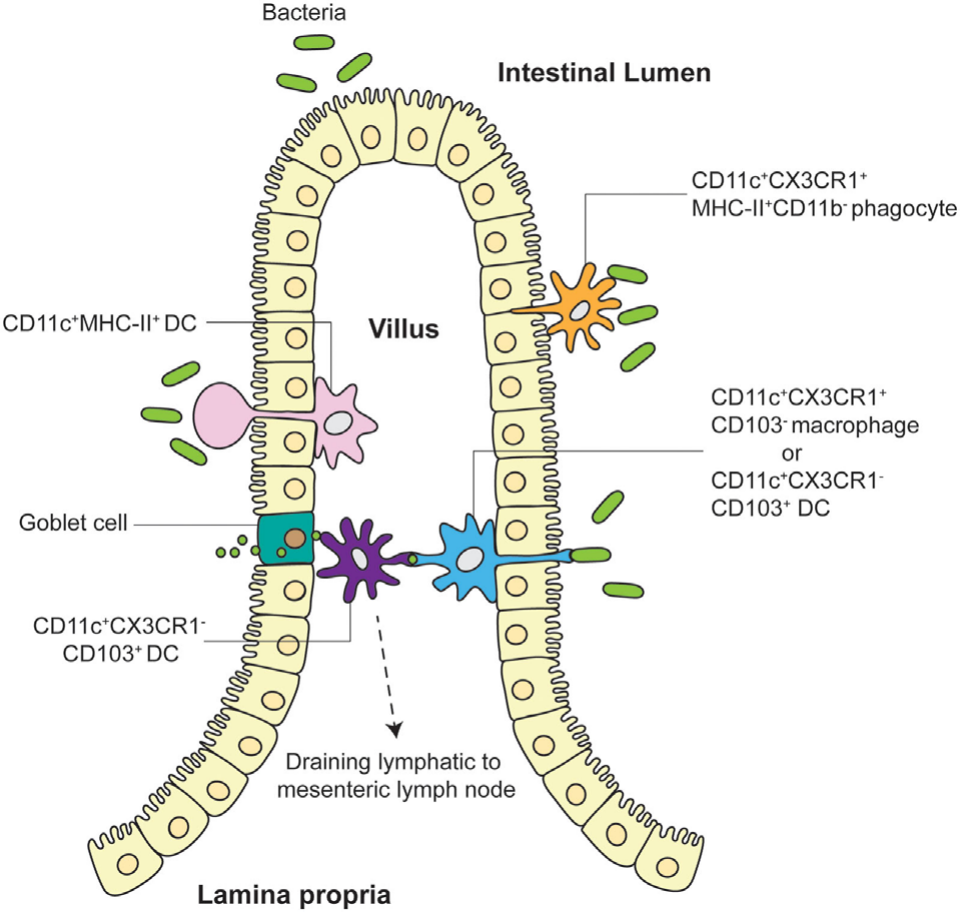
**Different models of TE and antigen uptake by LP APC**. LP APCs adjacent to the villus epithelia extend finger-like projections or balloon bodies (BB) directly into the gut lumen to sample microbes. Both CD11c^+^CX3CR1^+^CD103^−^ Macs and CD11c^+^CX3CR1^−^CD103^+^ DCs have been shown to push TEs into intestinal lumen to directly interact with pathogens. CD11c^+^MHC-II^+^ DCs have been visualized to extend BBs into the lumen in some explant preparations. Tight junction proteins expressed by LP APCs allow preservation of villus epithelia organization. CD103^+^ DCs closer to the villus core may receive antigen from goblet cells and/or CX3CR1^+^ Macs or may directly sample translocated pathogens/antigens themselves before migrating to the draining mesenteric LN. CD11c^+^CX3CR1^+^MHC-II^+^CD11b^−^ phagocytes have also been shown to unidirectionally translocate into the lumen to participate in pathogen exclusion.

Aside from mouse strain dependence, is there evidence for functional consequences of pathogen/antigen uptake by LP APCs *via* TEs? Schulz et al. have addressed this in studies elucidating the function of two major LP APC subpopulations, showing that the CX3CR1^+^ Macs population comprises non-migratory cells with limited capacity to prime naive T cells (23). In contrast, CD103^+^ LP DCs are fully capable of migrating to draining mesenteric LN and activating T cells. Anatomically, CX3CR1^+^ Macs were found adjacent to gut epithelia whereas CD103^+^ DCs were closer to the villus core. Previous data suggest that while CX3CR1^+^ Macs can efficiently uptake soluble antigen, CD103^+^ DCs do not share this ability but appear to be able to directly capture bacteria (21, 24). These data suggest that although CX3CR1^+^ “resident” LP Macs can uptake bacteria or antigen in the LP, they likely then pass it on to CD103^+^ “migratory” DCs, which then travel to the mesenteric LN for T cell activation. This model has been corroborated by Mazzini et al., who showed that CX3CR1^+^ Macs efficiently uptake antigen for transfer to CD103^+^ DCs in a process that is dependent on connexin 43 (25). Taken together, multiple avenues have been described for antigen uptake by LP DCs leading to antigen-specific T cell activation in the draining mesenteric LN: direct uptake of translocated pathogen/antigen, transfer of antigen from goblet cells or LP Macs, and direct sampling of pathogens *via* TEs (Figure 1). Gut APC TEs may therefore play important roles in initiating downstream gut-trophic adaptive immune responses, although further work is needed to better quantify TE functional contribution to the differential recognition and control of commensal and pathogenic microbiomes.

## Information Exchange Between LN Conduits and Follicular DCs (FDCs)

Transepithelial/transcompartmental extension behavior of DC and Macs is not unique to those found in the gut mucosa, as similar behavior can be found in secondary lymphoid organs. The LN is a highly specialized immune organ designed for the organization and fine tuning of immune cells in order to promote rapid and robust adaptive cell-mediated and humoral responses toward foreign insults. Antigen immigration into the LN parenchyma is done in an organized fashion directed mainly by APCs within the subcapsular sinus (SCS), medulla, and stromal cells that line the SCS and insulate the conduits in the LN. In 1965, Nossal and colleagues were the first to show that low molecular weight antigens could cross the vasculature of the LN capsule and contact immune cells within (26). They subcutaneously injected ^125^I-labeled intact flagella into the hind footpads of rats and then isolated the DLNs for scintillation counting (26). Two things pertaining to the LN follicles were observed. First, they observed a more rapid uptake of ^125^I-labeled flagella into LN follicles of rats that were previously immunized with unlabeled flagella (26). Second, the ^125^I-labeled flagella began to appear in cortical LN follicles within 30 min after injections and were retained for a week afterward (26). Nossal and colleagues attributed this appearance of ^125^I-labeled flagella in the follicles to what they called macrophage fibrils, which were also enhanced by opsonization of the ^125^I-labeled flagella bound by antibodies (26). In 1975, Arthur Anderson first introduced the concept that LNs contained traversing conduits through which antigens can travel. He and colleagues showed that low molecular weight substances could reach high endothelial venules (HEVs) in the LN paracortex as soon as 1 min after injection (27). Since then, studies of antigen transport within these conduits have blossomed, and they have revealed a complex filtering system in which specific immune cells, foreign- and self-antigens, and cytokines are shuffled, organized, and selected for entry into the LN parenchyma. Today, we identified these macrophage fibrils described by Nossal to be FDCs, which reside within B cell follicles. FDCs function by maintaining and organizing the B cell follicular zone (28) and aiding in the development of B cell germinal centers (2) through the release of cytokines and the presentation of unprocessed antigens to B cells in order to promote somatic hypermutation.

**Figure 2:**
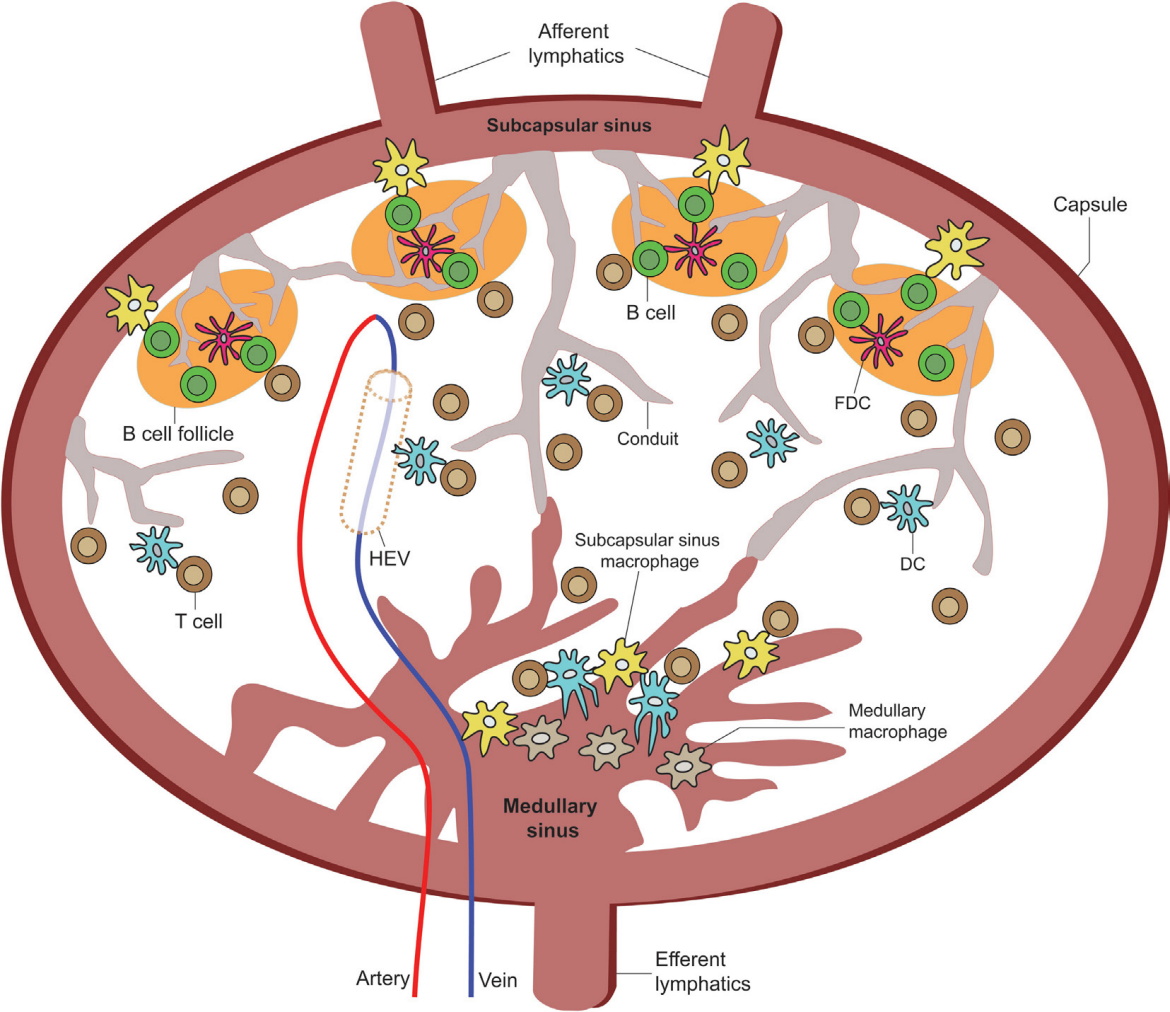
**Overview of LN APC positioning for TE sampling**. A cartoon of the LN depicting the various APC extensions into or in direct contact with the subscapular sinus, medullary sinus, B cell zone LN conduits, high endothelial venules, and paracortical zone LN conduits [adapted from Girard et al. (40)].

FDCs capture unprocessed, opsonized immune complexes (ICs) (29, 30), and free-form low molecular weight antigens [<70 kDa (5.5 nm)] (31, 32) for presentation to B cells through complement receptors 1 (CD21), 2 (CD35), and FcγRIIb (33–35). FDC capture of large ICs come primarily through B cell-antigen transference (Figure 2) (36–38), which can, in theory, be retained for months to years (39). However, the transport of small antigens from B cells to FDCs does not happen as efficiently as the transfer of larger ICs (38). In 2009, one study provided direct evidence that small antigens accumulating in the B cell follicles gained access by gaps between the fibroreticular stromal cells (FSC) network that are prominent in LN conduits within the B cell follicular zones (38). Adding to this, Bajénoff and Germain showed in 2009 that FDCs occupy a large portion of these gaps between FSCs, and FDCs are found to be in direct contact with LN conduits within the B cell follicles (31). To date, it is still unclear whether FDCs can capture antigen from within LN conduits by direct sampling or whether these low molecular weight antigens diffuse into the follicles and are subsequently captured by FDCs (39).

## LN-Resident and Migratory DCs Interact Directly with Lymphatic Conduits and HEVs

**Figure 3:**
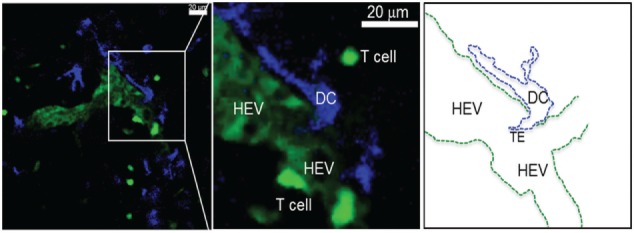
**Activated DCs reside near HEV after migrating from the periphery**. Single 0.5 μm optical section from an intravital 2PLSM experiment showing the perivascular juxtaposition of migrating DC (blue) from the periphery and the high endothelial venule (HEV; light green) in the LN. Newly i.v. injected T cells (deep bright green) can be seen attaching to the luminal wall as well as migrating in the LN parenchyma through the HEV structure (Alex Y. Huang). Intravital 2PLSM of mouse LN was performed under a protocol approved by the Case Western Reserve University IACUC.

The concept that resident DCs in the DLN can directly sample antigens coming from the lymph fluid was first documented by Manickasingham and Caetano Reis e Sousa (41) in 2000. They showed that before migratory DCs arrive at the DLNs, CD8α^+^/CD11c^+^ LN-resident DCs take up exogenous antigens delivered to the LN *via* the lymphatic fluid (41, 42). In 2003, Itano and colleagues showed that these resident DCs are primarily composed of MHC-II^high^/CD11c^+^/CD11b^+^/CD205^int^/CD8α^low^/B220^−^ interstitial DCs found in all LN types from the afferent ducts and MHC-II^high^/CD11c^+^/CD11b^+^/CD205^high^/CD8α^int^/B220^−^ epidermal Langerhans DCs, which migrate exclusively from the skin to DLNs (43). Sixt and colleagues provided direct evidence that immature resident myeloid (CD11b^+^/CD11c^+^) DCs are found to reside embedded within the cell layer of the FSC network located within the LN parenchyma and can make direct contact with the LN conduits though the expression of β1 integrins and the binding of laminin-10 and fibronectin on conduit fibers (1) (Figure 2). They also showed these DCs to be distinct from immigrating mature DCs, which do not associate with LN conduits but instead associate predominantly near HEVs (1, 40), where they can be observed to exhibit TE behavior into the HEVs (Figure 3; unpublished data). However, as in the case of FDC association with LN conduits, it is unclear how antigen is physically retrieved from the conduits by resident DCs, or whether such association licenses the DCs to exhibit TE behavior into the lymph fluid. Similarly, future studies are needed to establish immunological significance of trans-HEV dendritic extensions by DCs for immune cell recruitment or defense against blood-borne pathogens.

## CD169^+^ Macs Extend Processes from Subcapsular Sinus into LN Follicular Zones to Assist in Activating the Adaptive Humoral Response

Resident LN Macs can be divided into three main subsets based on their LN anatomical locations: SCS Macs, medullary sinus Macs, and medullary cord Macs (44). They originate from the bone marrow and can migrate to the LN under inflammatory and steady-state conditions by way of the afferent ducts (45). The function of each Mac population depends on the cues received from the surrounding LN microenvironment. Macs migrate to the LN by way of the afferent lymphatic duct; however, unlike DCs, they do not enter the LN parenchyma with the exception of a small subset of Macs (37, 44). While some Macs can transmigrate across the subcapsular floor into the LN follicles or the medullary cords, some Macs are embedded within the capsular linings of their respective areas and deliver antigens across compartments to the LN parenchyma (37, 46, 47). How exactly LN-bound Macs find themselves bound across the epithelial layers between the LN parenchyma and SCS remains to be fully explored, but evidence points toward chemokine signals, such as Lymphotoxin alpha-1/beta-2 (LTα_1_β_2_), secreted by B cells and other stimulating factors found within the afferent lymphatic ducts and around the LN follicles (44). Transluminal resident Macs can deliver both opsonized antigen in the form of ICs to non-cognate B cells by way of complement receptors 1 and 2 on the B cells for delivery to FDCs (37, 46) and processed antigens for delivery to DCs and T cells in the inter-follicular regions of the LN (Figure 2) (44). Therefore, intra-parenchymal Macs represent the first line of encounter against lymph-borne antigens and are capable of initiating the adaptive humoral immune responses.

## Local LN DCs Extend Dendritic Processes into Lymphatic Fluid to Initiate Early T Cell Activation

**Figure 4:**
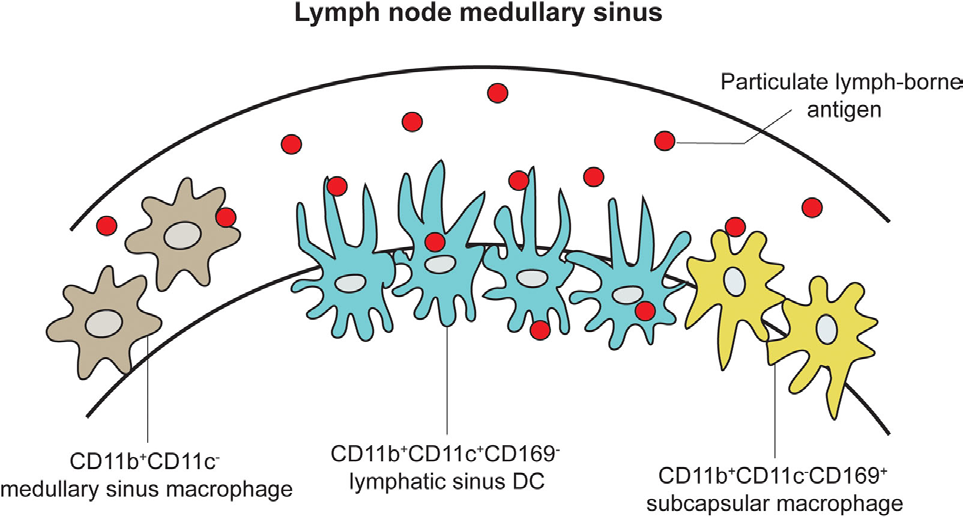
**LN DCs and Macs extend processes into the medullary sinus to directly sample lymph-borne antigen**. An expanded cartoon of the LN medullary sinus from Figure 2, depicting DCs, subcapsular, and medullary sinus macrophages embedded into the medullary sinus floor and contacting naive T cells [adapted from Gerner et al. (50)].

While Macs are observed to reach across the endothelial wall of the LN SCS and medullary sinus floors, migrating DCs have been implicated to induce structural changes of the SCS floor from within the afferent lymphatic duct to facilitate their transmigration across the SCS floor into LN parenchyma (48, 49). However, a recent study conducted by Gerner and colleagues revealed that DCs not only can transmigrate across the SCS but can also become embedded in the medullary sinus as resident DCs, extending their processes through intact LN sinus endothelium to sample and process antigens directly from the lymphatic fluid. In turn, this leads to the activation of T cells without the aid of migratory DC from the periphery (Figures 2 and 4) (50). Using real-time *in vivo* analysis through a combination of histocytometry, 2PLSM, and 3D LN imaging, Gerner and colleagues identified a subpopulation of DCs that are CD169^−^/CD11b^+^/CD11c^+^ and that transverse the LN medullary sinus. Using >70 kDa labeled proteins (to ensure that they cannot passively diffuse into the LN conduits) and both live and attenuated bacteria, they provided real-time data to demonstrate that these LN-resident DCs can indeed take up the antigens directly. Taking this a step further, they immunized OT-I and OT-II containing animals with conjugated OVA proteins with 1 μm microspheres to show OT-I and OT-II cell clustering around OVA beads-engulfed DC within 8 h. Additional experiments were performed to show that resident, not migratory, DCs accounted for this DC population.

## APC Extensions in Other Anatomical Compartments: Airway Lumen and CNS Vessels

**Figure 5:**
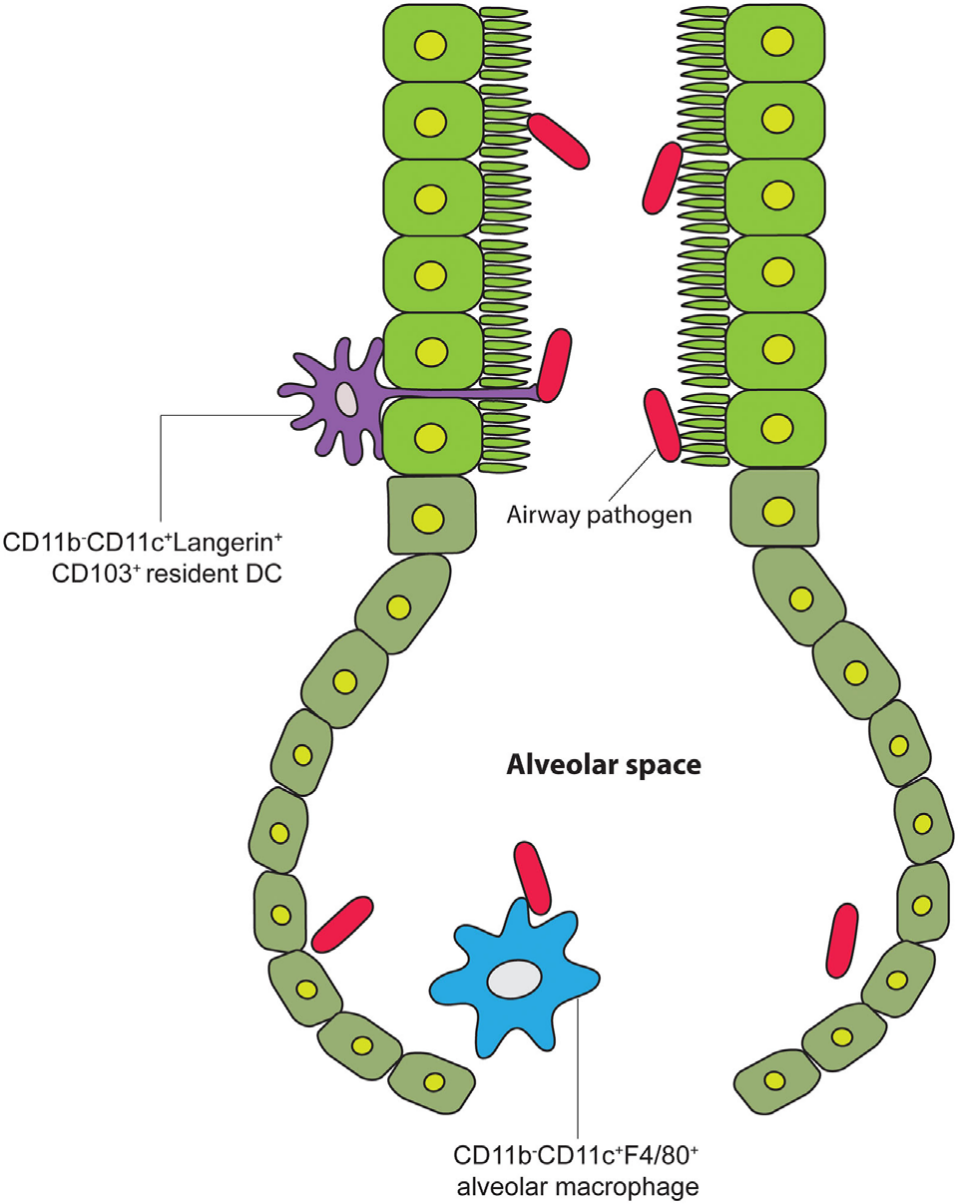
**Lung DCs sample airway pathogens *via* TEs**. A cartoon of the airway leading into the alveolar space, showing alveolar tissue-resident DCs extending their processes into the air canal between tight junctions of the alveolar epithelial cells to contact pathogens under steady-state conditions [adapted from Hammad et al. (60)].

Besides the gut mucosa and LN, DCs and Macs in other anatomical sites have been observed to exhibit transcompartmental reach across intact physical barriers (Figure 5). In 2006, two separate groups showed evidence in tracheal tissue explant and sections that CD103^+^ mucosal DCs expressed langerin and tight junction proteins that allowed them to migrate across the airway epithelia (51) and extend processes into the airway lumen to sample antigen (51, 52), which is consistent with findings of the same type of phenomenon found in the gut as discussed above. This was later corroborated by other studies using 2PLSM (53–55). This transluminal scanning behavior in the airway was shown to be directly tied to TLR4 signaling on the airway epithelial cells (56). However, despite the evidence that mucosa DCs can extend these processes into the airway lumen, the biological significance and contribution of this particular finding remains controversial. Zoltán Veres and colleagues used CD11c-enhanced yellow fluorescent protein transgenic mice to demonstrate both antigen capture and subsequent migratory behavior of airway mucosa DC populations (54). The movements of DCs were visualized in both *ex vivo* and *in vivo* preparations using 2PLSM. They showed that these DCs are able to process antigen, migrate to DLNs, and present to T cells; however, despite the ability of antigens to cross the airway epithelium and be taken up by CD11c^+^ alveolar macrophages in the lung parenchyma, visual evidence for airway DC’s ability to directly capture antigen through transepithelial extensions into the airway was documented only twice in multiple imaging experiments (54). The authors emphasize that these airway DC TE events were indeed very rare, making the functional relevance of these events in the airway difficult to discern (54). In stark contrast to the gut microenvironment, the CNS represents a unique, immune-privileged site wherein the resident APCs consist of microglia and perivascular/meningeal Macs (57, 58). As an area of the body that is sealed off by the blood–brain barrier, the CNS has historically not been thought of as a significant site of homeostatic antigen surveillance. Recently, however, it has been shown by intravital 2PLSM in the uninflamed CNS that CX3CR1^+^ APCs are able to extend dendrites into vessels to directly sample for antigen in the bloodstream (59). Interestingly, the number of these extensions per millimeter square of vessel wall significantly increases in the setting of EAE, while the numbers remain the same in the settings of dorsal column crush injury and intracranial tumor. These results suggest that inflammatory signals can drive intraluminal extensions of CNS APCs, which could then prime adaptive immune responses in an efficient *in situ* manner. The functional implication of this intravascular extension behavior by CNS resident CX3CR1^+^ APC remains to be fully elucidated.

## Concluding Remarks

Antigen-presenting cells are essential coordinators of adaptive immunity. At various anatomical sites, such as mucosal, vascular, and lymphatic interphases, they function as critical sentinels, constantly sensing the tissue microenvironment for information to help determine host response. Emerging bodies of evidence suggest a different view of their *in vivo* behavior than previously thought. Rather than positioning at mucosal, vascular, and lymphatic interphases with another tissue compartment passively awaiting encounters with invading pathogens, intravital imaging data provide a highly dynamic view of these important immune cells actively reaching across intact and separate anatomical compartments to survey potential offending agents on the other side of the physical barrier. More studies are required in the future to fully understand the functional implication of this common observation of APCs for potential therapeutic exploitation of this intriguing *in vivo* behavior.

## Author Contributions

FA, AT, and AH conceived the topic concept, performed literature review and critiques, and composed the entirety of the mini review content.

## Conflict of Interest Statement

The authors declare that the research was conducted in the absence of any commercial or financial relationships that could be construed as a potential conflict of interest.

## Abbreviations

2PLSM, two-photon laser scanning microscopy; APC, antigen-presenting cells; BB, balloon bodies; CNS, central nervous system; DC, dendritic cell; DLN, draining lymph node; FDC, follicular dendritic cell; FSC, fibroreticular stromal cell; HEV, high endothelial venule; IC, immune complex; LN, lymph node; LP, lamina propria; Mac, macrophages; MHC, major histocompatibility complex; PP, Peyer’s patch; SCS, subcapsular sinus; TCR, T cell receptor; TE, transepithelia/trans-mucosal extensions; TMD, trans-M dendrite.
